# Thermoregulation strategies in ants in comparison to other social insects, with a focus on red wood ants ( *Formica rufa* group)

**DOI:** 10.12688/f1000research.2-280.v2

**Published:** 2014-03-21

**Authors:** Štěpánka Kadochová, Jan Frouz

**Affiliations:** 1 Department of Ecology, Charles University, Prague, CZ12800, Czech Republic; 2 Institute for Environmental Studies, Charles University, Prague, CZ12800, Czech Republic

**Keywords:** review, wood ants, Formicinae, thermoregulation, metabolic heat production

## Abstract

Temperature influences every aspect of ant biology, especially metabolic rate, growth and development. Maintenance of high inner nest temperature increases the rate of sexual brood development and thereby increases the colony fitness. Insect societies can achieve better thermoregulation than solitary insects due to the former’s ability to build large and elaborated nests and display complex behaviour. In ants and termites the upper part of the nest, the mound, often works as a solar collector and can also have an efficient ventilation system. Two thermoregulatory strategies could be applied. Firstly the ants use an increased thermal gradient available in the mound for brood relocation. Nurse workers move the brood according to the thermal gradients to ensure the ideal conditions for development. A precise perception of temperature and evolution of temperature preferences are needed to make the correct choices. A second thermoregulatory strategy used by mound nesting ants is keeping a high temperature inside large nests. The unique thermal and insulation properties of the nest material help to maintain stable conditions, which is the case of the Wood ant genus *Formica*. Ants can regulate thermal loss by moving nest aggregation and alternating nest ventilation. Metabolic heat produced by ant workers or associated micro organisms is an important additional source of heat which helps to maintain thermal homeostasis in the nest.

## Introduction

Temperature is an important factor for all ectothermic organisms, including ants. Their rate of development is accelerated with high temperatures ^1^, the movement rate speeds up ^2^ and the rate of food and oxygen consumption also increases ^3^. Higher temperatures can be advantageous for colony fitness as it can increase reproduction rate though at the same time can be disadvantageous due to higher energy expenditure ^4^.

Most insect species have a solitary mode of life in adulthood; these individuals are able to regulate their body temperature through behavioural reactions such as sunning or seeking cool shelter ^5^. More sophisticated forms of thermoregulation can be found among social insects. They are able not only of regulating their own body temperature but also the temperature of the immediate surroundings. This ability is due to the large number of individuals in the society, their behavioural interactions and nest construction ^6^. The nest protects the whole colony and serves as a shelter for adults and, more importantly, as an incubator for the brood. Improved colony homeostasis could even be one of the reasons why insect sociality evolved ^5^. Thermoregulation, however, requires the expenditure of energy and so comes with costs as well as benefits.

The level of nest thermoregulation depends on many other factors, e.g. nest size, population size, and the moisture and thermal conductivity of the nest material. Among mound building ants two main thermoregulatory strategies can be distinguished: 1) moving the brood according to thermal gradients (natural or induced) and 2) keeping stable temperatures inside their nests. This paper’s objective is to provide an overview of nest thermoregulation strategies occurring among ant societies, with a focus on thermoregulation in the red wood ants ( *Formica rufa* group), in comparison to other social insects.

### Nest architecture and properties of nest material

Ants nest in wide range of materials; in soil, under stones, in leaf litter and even in living trees (see Table 1). Some of them build above-ground nests, usually from soil or organic material, called ant hills or mounds. These nests show an advanced ability in regulating inner temperature.

**Table 1.** Examples of thermoregulation and nesting strategies in social insects.

Thermoregulation characteristic	Thermoregulation strategy	Nest type	Taxa and species involved	References
Temperature is stable, same as the temperature of the soil	Brood translocation	Underground nests only	Ants: *Tetramorium* *tsushimae*, genus *Lasius* and *Myrmica*, *Pogonomyrmex*; Termites: genus *Coptotermes*	Hólldobler & Wilson (1990) ^23, 47^, Sanada - Morimura & al. (2005) ^59^
Temperature is not stable, nest follows microhabitat temperature - oscillations can be buffered by insulation properties of microhabitat, sometimes natural structures can be used as heat accumulators	Brood translocation	Nests in wood (logs or stumps), in leaf litter, and under stones	Ants: *Acromyrmex* *ambiguus, Camponotus* *mus, C. vicinus, Myrmica* *punctiventris Roger,* *Onychomyrmex hedleyi,* *Polyrhachis simplex* Termites: *Kalotermitidae,* *Hodotermitidae,* *Rhinotermitidae,* *Termopsidae*	Banschbach & al. (1997) ^32^, Bollazzi & Roces (2007) ^30^, Roces & Nunez (1989) ^29^, Chen & al. (2002) ^56^, Hólldobler & Wilson (1990) ^23, 47^, Miyata & al. (2003) ^58^, Ofer (1970) ^57^
Temperature oscillates, the thermal gradient could be greater than the ambient temperature because the mound surface serves as a solar collector	Brood translocation	Underground nest with aboveground crater or similar structure + above ground soil (soil and fecal pellets mixture) or thatch mound	Ants: *Acromyrmex heyeri,* genus *Lasius, Myrmica* *rubra, Pogonomyrmex* *occidentalis, Solenopsis* *invicta,* Termites: *Cephalotermes* *rectangularis,* *Microceretermes* *edantatus*, *Thoracotermes* *macrothorax*	Weir (1973) ^39^, Bollazzi & Roces (2002) ^38^, Cassil & al. (2002) ^69^, Cole (1994) ^60^, Hólldobler & Wilson (1990) ^23, 47^, Lüscher (1961) ^62^, Nielsen (1972) ^67^
Energy is accumulated by spatial structure - silk caps of pupal cells storing sun energy	Stable temperature	Paper nest	Hornet: *Vespa orientalis*	Ishay & Barenholz-Paniry (1995) ^64^
Energy is accumulated by sun bathing ants and is transported into the nest	Stable temperature	Underground nest + above ground organic mounds	Wood ants *: Formica rufa*, *F.polyctena*	Coenen-Stass (1985) ^14^, Frouz (2000) ^15^, Zahn (1958) ^51^
Nest is heated by the sun (temperature of peripheral layers oscillates) + inner core with more stable temperature, heated by metabolism of termites or ants	Stable temperature (?)	Aboveground nest in wood (living trees)	Termites: *Coptotermes* *acinaciformis, C. frenchi*	Greaves (1964) ^65^
	Stable temperature (?)	Underground nest + aboveground soil mound	Termites: family *Termitidae*, *Macrotermes bellicosus,* *Amitermes merionalis;* Ants genus *Atta - Atta texana,* *A. vollenweideri*	Kleineidam & al. (2001) ^27^, Hólldobler & Wilson (1990) ^23, 47^, Korb & Linsenmair (1998, 2000) ^25, 42^, Grigg (1973) ^26^
Nest is heated by sun (temperature of peripheral layers oscillates in small nests or stable in big ones) + inner core with stable temperature, heated by metabolism of ants + decay of organic material	Stable temperature	Underground nest + above ground organic mounds	Wood ants: genus *Formica* *– Formica aquilonia, F. rufa,* *F. polyctena*	Coenen-Stass (1985) ^14^, Frouz (2000) ^15^, Rosengren & al. (1987) ^46^, Zahn (1958) ^51^
Stable temperature inside the cluster is maintained by ant metabolism	Stable temperature	No stable nest - bivouacs	“Army ants” *Eciton burcheli,* *E. hamatum*	Franks (1989) ^48^, Schneirla (1971) ^41^
Active heating by members using contractions of muscles	Stable temperature	Wax or paper nest in air or various cavities	Honey bees, Stingless bees, Bumble bees, Wasps: *Apis mellifera, A. florea,* *A. dorsata, Bombus sp.,* *Trigona denoiti, Vespa* *simillima, V.xanthoptera,* *V. crabro*	Fletcher & Crewe (1981) ^61^, Heinrich (1981) ^20^, Ishay (1973) ^63^, Martin (1988) ^50^, Morse & Laigo (1969) ^66^, O´Donnell & Foster (2001) ^68^

The red imported fire ant *Solenopsis invicta* and members of the genus *Pogonomyrmex* build soil mounds from excavated soil ^7, 8^. These nests gain heat through solar radiation and the brood is moved along an increased thermal gradient ^1, 9^. Weaver ants from the genus *Oecophylla* construct their nest from living leaves, with the help of their own larvae, which produce a special “glue” from their salivary glands ^10^.

Red wood ants from the genus *Formica* build large nests from organic material that is based on a mixture of soil, twigs, coniferous needles and pebbles ^3, 11– 13^. In these nests a stable heat core can be maintained thanks to the good insulation properties of these materials and the metabolic heat produced by the ants or their associated microflora ^14, 15^.

The composition of the organic material is not the same throughout the whole nest volume ^3, 16^ and the mound structure is not rigid either. The ants loosen and renovate the nest structure and the organic material is continuously moved from the inside to the outer layers ^3^. Nest structure and architecture plays a vital role in nest thermoregulation. The tunnels and passages build by ants are important mainly for ventilation and humidity control, which affect the nest temperature. Pieces of tree resin are often incorporated into the *Formica* nest material because of its antimicrobial effect ^17^. The resin inhibits the growth of potentially pathogenic bacteria and fungi in the nest.

Thatch ants, *Formica obscuripes* ^18^ and *Acromyrmex heyeri* ^19^, use plant fragments as a building material and arrange them in a thick compact surface layer called “thatch” which has a lower thermal diffusivity than the surrounding soil. The thatch prevents nest overheating by the incoming solar radiation and avoids losses of the accumulated heat into the cold air during night ^19^.

Nest moisture can have two different and opposite thermoregulatory effects: 1) moisture can support microbial heat production (i.e. increase the temperature) ^3, 15^; 2) it can decrease the insulating properties of nest material (i.e. decrease the temperature) ^11^. A study of the relationship between daily temperature regime and moisture in *Formica polyctena* nests revealed two possible thermoregulation strategies which differ between dry nests and wet nests ^15^. Dry young nests are usually located in sunny open places ^12^. Solar radiation heats up the nest and keeps the nest material dry, with low heat capacity and conductivity. The thermal losses of dry nests are estimated to be 0.15–4.3 W per nest ^15^. The temperatures of dry nests are usually the highest in the evening and they drop during the night. Thermoregulation in dry nests is based on a combination of metabolic heating from the ants, the insulating properties of the nest and solar heating ^15^.

During forest succession, ant nests become more shaded and gradually switch to a wet thermoregulation regime ^12^. In the evening the temperature in these nests is low and it increases during the night. The high night temperature at the nest surface indicates substantial heat loss, about 24–30 W per nest. The wet nests have a high thermal capacity; increasing the temperature by 1°C requires a thermal input of 35 W ^15^. This means that maintaining a sufficient temperature in a wet nest requires a heat source beyond the metabolic heat produced by ants and the heat obtained from solar radiation. The additional heat source in wet nests is provided by microbial activity ^3, 15^. Both of these two types of thermoregulation strategies are applied in natural populations, but in different stages of nest development. The microbial community in *F. polyctena* nests differs from that in the surrounding soil in part because of differences in pH and food availability and quality ^13^. Thermoregulation via microbial heating was first proposed in 1915 by Wasmann ^3^. In 1980 the existence of microbial heating in *F. polyctena* mounds was confirmed in an experiment showing that in the absence of ants non-sterilized nest material generated a substantial amount of heat but sterilized nest material generated almost no heat ^3^.

Microbial activity can be estimated by calculating the respiration rate of the nest material, which is used as a proxy for the respiration rate of the microbes living in it ^3, 15^. There are detectable seasonal changes in the respiration rates of nest material with the highest rates found in summer. The mass-specific heat production of ants is higher than that of the nest material but, when considering the total mound volume, microbial heat production is more than seven times higher than the heat evolved by ants ^3^. Ants can affect microbial activity via nest material aeration, supply of fresh plant material and their own metabolic heat production. Since the microbial activity of wet nest material depends strongly on temperature ^3^, a temperature increase in some small parts of the nest (due to ant metabolism or sun radiation) results in an increase in microbial activity and consequently in a subsequent overall increase of nest temperature.

As first mentioned by Forel in the early 1920s the ant mound often serves as a solar collector ^20^. Solar energy can both increase the metabolism of ants and help heat the nest mound ^21^. Compared to underground nests, mounds absorb heat more quickly both in the direct sun and in the shade ^9^. Ants in the Northern Hemisphere usually remove shading grass from the south side of the mound so that the temperature increases quickly on that side. This creates a temperature gradient that many species use for brood displacement ^9^. Other species decorate the mound surface with small pebbles or dead vegetation, which can work as heat collectors or as radiation reflectors ^22^.

Mounds of some *Formica*, *Solenopsis* and *Lasius* species are asymmetric, with the main axis oriented in a south-north direction ^23, 24^. In *S. invicta* the angle of the south slope of the mound is negatively correlated with the maximal sun angle ^22^. Sun-influenced thermoregulation in termites has also been documented, for example in fungus-growing members of the genus *Macrotermes* ^25^. The amount of intercepted sunlight influences the shape of termite mounds and leads to great structural differences in nests in forests vs. savannahs ^25^. But the most admirable sun-induced nest shape differences can be seen in Australian “magnetic termites” *Amitermes meridionalis* ^26^. The nests are wedge-shaped with apparent north-south orientation which prevents overheating at noon and enables maintaining the warmth of nest in the evening.

Effective ventilation takes place as part of nest thermoregulation in many ant species, being regulated by the opening and closing of nest entrances (see below). A ventilation system in nests of the leaf-cutting ant genus *Atta* was described by Kleineidam *et al.* ^27^. There is not a thermal gradient big enough to generate thermal convection flow; rather the ventilation in *Attini* nests is driven by the wind. There are many openings on the nest surface, which are functionally divided into entry and exit tunnels. Wind flowing over the nest from any direction causes air to exit from the central tunnel and to enter tunnels at the nest periphery ^27^. This ensures exchange of respiratory gasses and optimal thermal conditions for symbiotic fungi which are damaged by temperatures higher than 30°C ^28^.

### Behavioural reactions of ant workers

Behavioural reactions of ants are based on sensing temperature and temperature preferences ^5^. To react to these gradients, ants have evolved inherent temperature preferences, which are the key element in thermoregulatory behaviour ^4, 29^. Nurse workers are able to choose the optimal temperature for pupae production and sexual brood incubation and to move the brood along temperature and humidity gradients to achieve the best conditions for its development ^5, 29, 30^.

The optimal temperature range is variable for different groups of social insects, for example the brood of the honey bee *Apis mellifera* develops fastest at 35°C ^31^. In *Formica polyctena* a temperature 29°C is preferred for pupal development ^14^, whilst colonies of *Solenopsis invicta* can grow only between 24 and 36°C ^1^. In contrast the genus *Myrmica* is adapted to cold climates, *M. rubra* ^4^ and *M. punctiventris* ^32^ prefer temperatures between 19 and 21°C, about 8°C lower than the temperature preferred by other ants.

Temperature preferences can be affected by many factors including age and sex ^33^, working caste or feeding condition ^1^, or prior acclimation ^34^. Ant queens in *Formica polyctena* ^35^ and *Solenopsis invicta* ^1^ prefer slightly higher temperatures than workers, especially during the egg-laying phase; inactive queens may prefer cooler temperatures. Workers generally prefer lower temperatures, which decrease their metabolic rate and increase their lifespan ^1, 36^. A decrease of 2°C can lengthen the worker lifespan in *S. invicta* by 14% ^37^. Preferences of nurse workers tend to be shifted towards the higher temperatures that favour brood development ^1, 4, 21, 29^. Similar patterns have also been found in *Apis mellifera* ^5, 31^.

Most ant species rely on brood translocation along temperature gradients as the main thermoregulatory strategy ^6, 9, 23, 29, 30, 38^. The brood translocation usually has characteristic time rhythms, for example *Camponotus mus* follow a photoperiodic circadian rhythm. In the presence of a temperature gradient, nurse workers move the brood twice each day ^29^. The first displacement starts at 2 pm, when the brood is transported from the colder night location to a warmer day location. This movement occurs 6 h after sunrise, i.e., in the middle of photophase. At 10 pm (8 h after the first displacement and 2 h after nightfall), the brood is transported back to the night location. Under artificial light/dark day cycles the brood translocation rhythm changes according to the new photophase length ^29^. In *Solenopsis invicta* moving the brood up and down along temperature gradients does not seem to depend on the time of day or photoperiod ^9^.

In response to temperature gradients, leaf-cutting ants from the genus *Acromyrmex* move not only the brood but also the symbiotic fungi which provide their food. The fungus requires high humidity and temperatures between 25 and 30°C ^28^. *Acromyrmex ambiguus* workers move the fungus garden according to humidity conditions, but they are also capable of nest humidity regulation by changing nest architecture. The flow of dry air into the colony is a signal for workers to plug ventilation tunnels to prevent nest from drying out ^38^. Similar behaviour has been proposed for termites ^39^.

When the nest interior becomes too hot, workers can reduce the inner temperature in several ways. In ants and termites nest cooling is usually achieved by changes in building behaviour. Workers of leaf-cutting ants in the genera *Atta* and *Acromyrmex* open tunnels to allow air circulation ^27, 30, 38^; this behaviour could be limited by a trade off for humidity control ^40^. Ants of the genus *Formica* can also partly remove the nest material, which reduces the wall thickness and increases heat dissipation ^16^. In the genus *Eciton*, loosening of bivouacs´ structure (temporary nests similar to a honeybee swarm) is an effective way of cooling ^41^.

Even more efficient ventilation systems providing both temperature regulation and respiratory gas exchange can be found in nests of the termite *Mactorermes bellicose*. These nests can have either externally or internally driven ventilation, depending on the habitat, nest shape (cathedral vs. dome shaped nest) or the time of day ^42^. Cathedral shaped nests in open habitats are warmed by the sun, which creates a steep thermal gradient leading to convention currents in peripheral air tunnels. Dome shaped nests located in the forest rely more on internally driven ventilation. The same is true for cathedral nests at night ^42^.

In contrast, bees, wasps and bumblebees cool their nests by wing-fanning and regurgitating water droplets. Water is spread over the brood comb surface enabling cooling through evaporation ^5^. Cooling by water evaporation is very effective. Lindauer ^43^ placed a bee hive onto a lava plain where the surface temperature reached 70°C. Taking water ad libitum, the bees were able to maintain the hive temperature at the favoured 35°C.

Some social insect species have evolved highly elaborated systems of thermoregulation that enable to keep stable temperatures inside their nests. Bees, bumblebees and wasps are capable of direct incubation of selected pupae. Specialized workers sit on the surface of the brood cell and maintain their thoracic temperature over 35°C. Bees sitting inside empty brood cells can heat six brood cells at one time ^44^. Metabolic heat can also be used for protection, as shown in the interaction between the predatory hornet *Vespa mandarina japonica* and Japanese honeybees *Apis cerana japonica* ^45^. If the hornet attacks a honeybee nest it is surrounded by bee workers who increase their body temperature to a level which is lethal for the hornet but not for the honeybees ^45^.

Although ants cannot actively produce heat they are able to use indirect metabolic heat (i.e. heat produced as a by-product of metabolism) for ensuring brood development. This ability has so far been postulated for the *Formica rufa* group ^3, 15, 46^ and the army ant genus *Eciton* ^41, 47, 48^.

The Neotropical army ants *Eciton hamatum* and *Eciton burchelli* form temporary swarms called bivouacs, which can regulate temperature very precisely to ensure optimal conditions for developing brood ^41^. According to Franks ^48^ bivouacs have a similar construction to a bee swarm; they can be divided into an outer mantel and inner core, which together maintain a stable temperature between 27.5 and 29.5°C regardless of the ambient temperature. On cold days the bivouacs change shape: they become more hemispherical to reduce the surface to volume ratio ^48^. Franks ^48^ postulates that all the heat required by the bivouac can be produced by ant metabolism.

Red wood ants of the Palearctic genus *Formica* ( *Formica rufa* group) are supposed to use metabolic heat to maintain a heat core, an area with high and stable temperatures, in their nests. High temperatures are required for sexual brood development; nests producing sexual offspring always have higher temperatures than those producing only workers ^46^. The heat core’s position moves according to nest shape and size ^3, 15^. The temperature inside nests of red wood ants begins to increase very early in the spring, even when the nest surface is covered by ice and snow ^46^. At this time some nests can contain larvae, pupae, and even some winged individuals, indicating that the inner heating must have started much earlier, because larval development cannot start in a cold nest and requires some weeks of constant warm temperature ^21^. It is supposed that in large nests of *F. rufa* containing over 1 million workers, spring nest heating can start as an autocatalytic process ^46^ that relies on utilizing lipid reserves in young workers ^50^.

Another factor contributing to quick spring increase of nest temperature in red wood ants is a temperature intake by ant bodies. The *Formica* ants are dark colored so they heat up quickly when exposed to the sun during their outside-nest activities. In the spring ants are observed to create clusters on the mound surface as they bask in the sun ^51^. Their bodies contain a substantial amount of water which has high thermal capacity making ant bodies an ideal medium for heat transfer. After getting hot enough the ants move inside the nest where the accumulated heat is released. This principle works throughout the year but in spring it is most obvious and supported by ant clustering on the nest surface ^15, 46^.

Daily temperature fluctuations in the red wood ants nest seem to be correlated with temperature-dependent changes in ant density and ant aggregations in the nest. The highest nest temperatures usually occur in the afternoon or in the evening which corresponds with the return of foragers ^15, 46^. This apparently results from the heat brought into the nest by returning workers (heat coming from absorbed solar energy) as well as the heat generated by worker metabolic heat production within the nest. In some nests the temperature drops slightly in the morning when ants leave the nest ^15, 16^. Heat coming from the metabolism of foragers clustered in the nest center on cool days, when ambient temperature limits outdoor activities, could also explain a negative correlation between the inner nest temperature and the ambient temperature found occasionally in spring ^46^.

The seasonal fluctuations in the thermoregulation behaviour of *Formica polyctena* along a geographic gradient were studied by Frouz & Finer ^49^. Both in Finland and the Czech Republic the ant colonies maintained a high nest temperature (over 20°C) in spring and summer, for about 65–129 days. A rapid increase in the inner nest temperature in early spring was observed, mostly at the beginning of April. Annual nest temperature peaked in June in both locations and decreased gradually from August to November. Nest temperatures fluctuated more in the Czech Republic than in Finland, possibly because of greater differences between day and night ambient temperatures ^49^.

An interesting question is why *F. polyctena* maintain stable nest temperatures for the same period in Finland and the Czech Republic even though the length of the vegetation season and the ambient temperatures in Finland and Czech Republic are different. This might be explained by the reproduction cycle of the queen. Regular shifts between reproduction and diapauses has been documented in *F. polyctena* queens. The queen enters diapauses after 100 days of reproduction even at a constant temperature and photoperiod ^35, 52^. It was postulated that ants maintain high temperatures only during the queen’s reproduction phase ^49^. After that the nest temperature drops, despite the fact that the outside temperature usually does not limit foraging and the ants are still active.

### Use of metabolic heat for nest thermoregulation in ants

Maintenance of a high inner nest temperature has been observed in ants, especially in species which build above-ground nests or inhabit tree hollows ^15, 46, 53^. Heating of these structures is much easier than heating underground nests, because the surrounding soil has a large heat capacity and conductivity.

In moderate climates most ants build nests in the soil where the temperature is quite stable ( Table 1) or on the soil surface under a layer of leaf litter where the temperature can be buffered by the insulating properties of the nest material. Many species in the Northern Hemisphere also nest under rocks or stones which serve as heat collectors ( Table 1). In the tropics only a few species nest in soil and the majority of species inhabit small pieces of rotting wood ^5^. More precise microclimate regulation is achieved in the mound-building species of the genera *Atta*, *Acromyrmex*, *Myrmicaria*, *Pogonomyrmex*, *Solenopsis*, *Iridomyrmex*, *Formica*, and *Lasius* ( Table 1).

The maintenance of a stable temperature in red wood ant genus *Formica* nests during spring and summer is widely known and has been the subject of many studies ^12, 16, 46, 49, 53^. These ants are able to maintain thermal homeostasis in the nest because of the insulation and heat storage provided by the nest material ^15, 20^. Mound size is generally correlated with the number of inhabitants ^54, 55^. In small nests located in sunny areas the solar radiation and the insulation properties of the nest material are thought to be the key elements in nest thermoregulation ^11, 12^. In larger nests there are internal sources of heat production, such as ant and microbial metabolic heat, that enable the maintenance of high inner temperatures even in a permanently cold environment ^46, 49^.

Many other ant species (see Table 1) build hill-shaped nests from soil. These nests show large spatiotemporal variations in temperature and ants select the optimal temperature for brood development by brood displacement ^1^. These nests, however, do not have a stable heat core. The heat core where the temperature is stable and higher than ambient temperature for an extended period of time (several months) can be found only in a minority of ant species. Why is this the case?

The heat core exists in the nests/hives of winged insects that are capable of active heating, i.e. bees, wasps, bumblebees (thermogenesis in flying muscles), and some ants, especially the genus *Formica* ^3, 15, 46^, the army ant genus *Eciton* ^41, 47, 48^ and probably in fungus-growing termites ^42^. But there is no evidence of a stable heat core in *Solenopsis invicta* or in *Acromyrmex heyeri*, which also build upper-ground hills from materials with suitable insulation properties such as soil or dead vegetation. As discussed earlier, thermoregulation can also be the result of nest architecture, and only those species that build nests with a low thermal heat capacity and a low thermal conductivity are likely to maintain a heat core. In summary, the use of metabolic heat production for maintenance of a heat core depends on the insulation properties of the nest and the size of the individual workers and of the entire colony.

The latest review about nest thermoregulation in social insects ^6^ distinguished three types of ant thermoregulation strategies: “First, like many social bees, some ant species rely on protection from a cavity, such as a tree stump or underground burrows ^56^. Second some migrate their nest frequently, varying the amount of cover they select, depending on the temperature and season ^57, 58^. Third some others move their brood to areas of optimal temperature within the same nest” ^29^. We suggest dividing the last category in the following way: a) ants that move the brood in daily cycles to places with optimal temperatures within the whole nest structure, for example *Solenopsis invicta* ^9^ or *Camponotus mus* ^29^ b) ants that keep a stable heat core inside their nest and do not move the brood from the nest interior, including the ants from *Formica rufa* group ^3, 15^.

## Conclusion

Despite a wide variety of thermoregulatory strategies observed among ant societies some general trends can be found. Two opposite thermoregulatory strategies in mound building ants could be distinguished. Firstly nests with low insulative properties which work as solar collectors and thus increase the available thermal gradient for brood development. Secondly nests which steadily maintain higher inner temperature thanks to low thermal conductivity of the nest material, good insulative properties and metabolic heat produced by ants or associated micro organisms. An example of this strategy is seen in the *Formica rufa* ant group.

## References

[1] Porter SD Tschinkel WR: Fire ant thermal preferences: behavioral control of growth and metabolism. *Behav Ecol Sociobiol.* 1993;32(5):321–329 10.1007/BF00183787

[2] Challet M Jost C Grimall A: How temperature influences displacements and corpse aggregation behaviors in the ant *Messor sancta*. *Insect Soc.* 2005;52(4):309–315 10.1007/s00040-005-0821-1

[3] Coenen-Stass D Schaarschmidt B Lamprecht I: Temperature distribution and calorimetric determination of heat production in the nest of the wood ants *Formica polyctena (Hymenoptera Formicidae).* *Ecology.* 1980;61:238–244 10.2307/1935180

[4] Brian MV: Temperature choice and its relevance to brood survival and caste determination in the ant *Myrmica rubra L.* *Physiol Zool.* 1973;46(4):245–252 Reference Source

[5] Wilson EO: The insect societies. Belknap Press of Harvard University Press, Massachusetts.1971;548 Reference Source

[6] Jones JC Oldroyd BP: Nest Thermoregulation in Social Insects. *Adv in Insect Phys.* 2006;33:153–191 10.1016/S0065-2806(06)33003-2

[7] Porter SD: Impact of temperature on colony growth and developmental rates of the ant *Solenopsis invicta.* *J Insect Phys.* 1988;34(12):1127–1133 10.1016/0022-1910(88)90215-6

[8] Carlson SR Whitford WD: Ant mound influence on vegetation and soils in semiarid mountain ecosystem. *American Midland Naturalist.* 1991;126(1):125–139 Reference Source

[9] Penick CA Tschinkel WR: Thermoregulatory brood transport in the fire ant *Solenopsis invicta.* *Insect Soc.* 2008;55(2):176–182 10.1007/s00040-008-0987-4

[10] Hölldobler B: Territorial Behaviour in the Green Tree Ant ( *Oecophylla smaragdina*). *Biotropica.* 1983;15(4):241–250 10.2307/2387648

[11] Brandt CJ: The thermal diffusivity of the organic material of a mound of *Formica polyctena* Foerst in relation to the thermoregulation of the brood (Hymenoptera, Formicidae). *Neth J Zool.* 1980;30(2):326–344 10.1163/002829679X00449

[12] Kilpeläinen J Punttila P Finér L: Distribution of ant species and mounds (Formica) in different-aged managed spruce stands in eastern Finland. *J Appl Entomol.* 2008;132(4):315–325 10.1111/j.1439-0418.2007.01244.x

[13] Frouz J Kalčík J Cudlín P: Accumulation of phosphorus in nests of red wood ants *Formica s. str.* *Ann Zool Fennici.* 2005;42(3):269–275 Reference Source

[14] Coenen-Stass D: Zum Verhalten der roten Waldameise, *Formica polyctena.* (Hymenoptera, Formicidae) im Klimagradient während der Brutpflege. *Verhandlungen der Deutschen Zoologischen Gesellschaft.* 1985;78:204–112 Reference Source

[15] Frouz J: The effect of nest moisture on daily temperature regime in the nests of *Formica polyctena* wood ants. *Insect Soc.* 2000;47(3):229–235 10.1007/PL00001708

[16] Horstmann K Schmid H: Temperature regulation in nests of the wood ant, Formica polyctena (Hymenoptera: Formicidae). *Entomologia Generalis.* 1986;11(3–4):229–236 10.1127/entom.gen/11/1986/229

[17] Castella G Chapuisat M Christe P: Prophylaxis with resin in wood ants. *Anim Behav.* 2008;75(4):1591–1596 10.1016/j.anbehav.2007.10.014

[18] Mciver JD Torgersen TR Cimon NJ: A supercolony of the thatch ant *Formica obscuripes Forel* (Hymenoptera: Formicidae) from the Blue Mountains of Oregon. *Northwest Sci.* 1997;71(1):18–29 Reference Source

[19] Bollazzi M Roces F: The thermoregulatory function of thatched nests in the South American grass-cutting ant, *Acromyrmex heyeri.* *J Insect Sci.* 2010;10(137):1–17. 10.1673/031.010.13701 20883129 PMC3016983

[20] Seeley TD Heinrich B: Regulation of temperature in the nest of social insects. In: Insect Thermoregulation (HEINRICH, B. Ed.). John Wiley and Sons, Inc,.1981;pp. 160–234.

[21] Kneitz G: Versuche zur Wärmeorientierung von Arbeiterinnen der Waldameisenart *Formica polyctena* Foerst. (Hymenoptera, Formicidae). *Insect Soc.* 1966;13:285–296 10.1007/BF02222388

[22] Vogt JT: Quantifying imported Fire ant (Hymenoptera: Formicidae) mounds with airborne digital imagery. *Environ Entomol.* 2004;33(4):1045–1051 10.1603/0046-225X-33.4.1045

[23] Hölldobler B Wilson EO: The Ants. The Belknap Press of Harvard University Press, Cambridge,1990;732 pp Reference Source

[24] Vogt JT Wallet B Freeland TB Jr: Imported fire ant (Hymenoptera: Formicidae) mound shape characteristics along a north-south gradient. *Environ Entomol.* 2008;37(1):198–205. 10.1603/0046-225X(2008)37[198:IFAHFM]2.0.CO;2 18348811

[25] Korb J Linsenmair KE: The effects of temperature on the architecture and distribution of *Macrotermes bellicosus* (Isoptera, Macrotermitinae) mounds in different habitats of a West African Guinea savanna. *Insec Soc.* 1998;45(1):51–65 10.1007/s000400050068

[26] Grigg GC: Some consequences of the shape and orientation of 'magnetic' termite mounds. *Aust J Zool.* 1973;21(2):231–237 10.1071/ZO9730231

[27] Kleineidam C Ernst R Roces F: Wind induced ventilation of the giant nests of the leaf-cutting ant *Atta vollenweideri.* *Naturwissenschaften.* 2001;88(7):301–305. 10.1007/s001140100235 11544898

[28] Powell RJ Stradling DJ: Factors influencing the growth of the *Attamyces bromatificus,* a symbiont of Attine ants. *Trans Br Mycol Soc.* 1986;87(2):205–213 10.1016/S0007-1536(86)80022-5

[29] Roces F Núñez JA: Brood translocation and circadian variation of temperature preference in the ant *Campotonus mus.* *Oecologia.* 1989;81(1):33–37 10.1007/BF00377006 28312153

[30] Bollazzi M Roces F: To build or not to build: circulating dry air organizes collective building for climate control in the leaf-cutting *ant Acromyrmex ambiguus.* *Anim Behav.* 2007;74(5):1349–1355 10.1016/j.anbehav.2007.02.021

[31] Jones JC Myerscough MR Graham S: Honey bee nest thermoregulation: diversity promotes stability. *Science.* 2004;305(5682):402–404. 10.1126/science.1096340 15218093

[32] Banschbach VS Levit N Herbers JM: Nest temperatures and thermal preferences of a forest ant species: is seasonal polydomy a thermoregulatory mechanism? *Insect Soc.* 1997;44(2):109–122 10.1007/s000400050034

[33] Cokendolhper JC Francke OF: Temperature preferences of four species of the fire ants ( *Hymenoptera: Formicidae: Solenopsis*). *Psyche.* 1985;92(1):91–101 10.1155/1985/32878

[34] Coenen-Stass D: Untersuchungen fiber die jahreszeitlichen Klimaprfiferenz der roten Waldameise *Formica polyctena* (Hymeno-ptera, Formicidae).Mitteilungen der Deutschen Gesellschaft für Allgemeine und Angewandte Entomologie.1987; **5**:44–48.

[35] Kipyatkov VE Schederova SS: Seasonal changes in behavior patterns of the ant *Formica polyctena* in an artificial nest with temperature gradient. *Zool Zhurnal.* 1985;65(12):1847–1857 Reference Source

[36] Ceusters R: Social homeostasis in colonies of *Formica polyctena* Foerst. (Hymenoptera, Formicidae): nest form and temperature preferences. Proc. 8th Int. Cong, International Union for the Study of Social Insects. Wageningen,1977;pp. 111–112 Reference Source

[37] Calabi P Porter SD: Worker longevity in the fire ant *Solenopsis invicta*: Ergonomic considerations of correlations between temperature, size and metabolic rates. *J Insect Physiol.* 1989;35(8):643–649 10.1016/0022-1910(89)90127-3

[38] Bollazzi M Roces F: Thermal preference for fungus culturing and brood location by workers of the thatching grass-cutting ant Acromyrmex heyeri. *Insect Soc.* 2002;49(2):153–157 10.1007/s00040-002-8295-x

[39] Weir J: Air flow, evaporation and mineral accumulation in mounds of *Macrotermes subhyalinus.* (rambur). *J Anim Ecol.* 1973;42(3):509–520 10.2307/3120

[40] Bollazzi M Roces F: Leaf-cutting ant workers ( *Acromyrmex heyeri*) trade off nest thermoregulation for humidity control. *J Ethol.* 2010;28(2):399–403 Japan Ethological Society and Springer. 10.1007/s10164-010-0207-3

[41] Schneirla TC: Army Ants: a Study in Social Organization (TOPOFF, H. Ed.). Freeman, San Francisco.1971 Reference Source

[42] Korb J Linsenmair KE: Thermoregulation of termite mounds: What role does ambient temperature and metabolism of the colony play? *Insect Soc.* 2000;47(4):357–363 10.1007/PL00001731

[43] Lindauer M: Temperaturregulierung und Wasserhaushalt im Bienenstaat. *Z Vergl Physiol.* 1954;36(4):391–432 10.1007/BF00345028

[44] Kleinhenz M Bujok B Fuchs S: Hot bees in empty broodnest cells: heating from within. *J Exp Biol.* 2003;206(Pt 23):4217–4231. 10.1242/jeb.00680 14581592

[45] Ono M Igarashi T Ohno E: Unusual thermal defense by a honeybee against mass attack by hornets. *Nature.* 1995;337(6547):334–336 10.1038/377334a0

[46] Rosengren R Fortelius W Lindström K: Phenology and causation of nest heating and thermoregulation in red wood ants of the *Formica rufa* group studied in coniferous forest habitats in southern Finland. *Ann Zool Fennici.* 1987;24:147–155 Reference Source

[47] Hölldobler B Wilson EO: The Ants. The Belknap Press of Harvard University Press, Cambridge,1990;732 pp 10.1126/science.248.4957.897

[48] Franks NR: Thermoregulation in army ant bivouacs. *Physiol Entomol.* 1989;14(4):397–404 10.1111/j.1365-3032.1989.tb01109.x

[49] Frouz J Finer L: Diurnal and seasonal fluctuations in wood ant *(Formica polyctena*) nest temperature in two geographically distant populations along a south-north gradient. *Insect Soc.* 2007;54(3):251–259 10.1007/s00040-007-0939-4

[50] Martin SJ: Thermoregulation in *Vespa simillima xanthoptera.* (Hymenoptera, Vespidae). *Kontyu.* 1988;56(3):674–677 Reference Source

[51] Zahn M: Temperatursinn, Wärmehaushat und Bauweise der role Waldameisen ( *Formica rufa* L). *Zoologische Beitraege.* 1958;3:127–194.

[52] Kipyatkov VE Schederova SS: The endogenous rhythm of queens' reproductive activity in red wood ants ( *Formica* group). *Zool Zhurnal.* 1990;69(5):40–52 Reference Source

[53] Raignier A: L’économie thermique d’une colonie polycalique de la fourmi des bois ( *Formica rufa polyctena* Foerst). *La Cellule.* 1948;51:289–368 Reference Source 18910548

[54] Tschinkel WR: Seasonal life history and nest architecture of a winter-active ant, *Prenolepis imparis.* *Insect Soc.* 1987;34(3):143–164 10.1007/BF02224081

[55] Chen YH Robinson EJH: A comparison of mark-release-recapture methods for estimating colony size in the wood ants *Formica lugubris.* *Insect Soc.* 2013;60(3):351–359 10.1007/s00040-013-0300-z

[56] Chen Y Hansen LD Brown JJ: Nesting sites of the carpenter ant, *Camponotus vicinus* (Mayr) (Hymenoptera: Formicidae) in northern Idaho. *Environ Entomol.* 2002;31(6):1037–1042 10.1603/0046-225X-31.6.1037

[57] Ofer J: *Polyrhachis simplex*, the weaver ant of Israel. *Insect Soc.* 1970;17(1):49–81 10.1007/BF02223772

[58] Miyata H Shimamura T Hirosawa H: Morphology and phenology of the primitive ponerine army ant *Onychomyrmex hedleyi* (Hymenoptera: Formicidae: Ponerinae) in a highland rainforest of Australia. *J Nat Hist.* 2003;37(1):115–125 10.1080/713834393

[59] Sanada-Morimura S Satoh T Obara Y: Territorial behavior and temperature preference for nesting sites in a pavement ant *Tetramorium tsushimae.* *Insect Soc.* 2006;53(2):141–148 10.1007/s00040-005-0849-2

[60] Cole BJ: Nest architecture in the Western harvester ant, *Pogonomyrmex occidentalis* (Cresson). *Insect Soc.* 1994;41(4):401–410 10.1007/BF01240643

[61] Fletcher DJC Crewe RM: Nest structure and thermoregulation in the stingless bee *Trigona* ( *Plebeina*) *denoiti Vachal* (Hymenoptera: Apidae). *J Entomol Soc South Afr.* 1981;44(2):183–196 Reference Source

[62] Lüscher M: Air-conditioned termite nests. *Sci Am.* 1961;205:138–145 10.1038/scientificamerican0761-138

[63] Ishay J: Thermoregulation by social wasps: behavior and pheromones. *Trans N Y Acad Sci.* 1973;35(6):447–462. 10.1111/j.2164-0947.1973.tb01518.x 4516097

[64] Ishay JS Barenholz-Paniry V: Thermoelectric effect in hornet ( *Vespa orientalis*) silk and thermoregulation in a hornet’s nest. *J Insect Physiol.* 1995;41(9):753–759 10.1016/0022-1910(95)00034-R

[65] Greaves T: Temperature studies of termite colonies in living trees. *Aust J Zool.* 1964;12(2):250–262 10.1071/ZO9640250

[66] Morse RA Laigo FM: *Apis dorsata* in the Philippines. Monogr. *Philipp Assoc Entomol.* 1969;1:1–96 Reference Source

[67] Nielsen MG: An attempt to estimate energy flow through a population of workers of *Lasius Alienus.* (forst), (Hymenoptera: Formicidae). *Natura Jutlandica.* 1972;16:99–107 Reference Source

[68] O´Donnell S Foster RL: Thresholds of response in nest thermoregulation by worker bumble bees, *Bombus bifarius nearcticus* (Hymenoptera: Apidae). *Ethology.* 2001;107(5):387–399 10.1046/j.1439-0310.2001.00668.x

[69] Cassill DL Tschinkel WR Vinson SB: Nest complexity, group size and brood rearing in the fire ant, *Solenopsis invicta*. *Insect Soc.* 2002;49(2):158–163 10.1007/s00040-002-8296-9

