# Supplementary material for: Thermoregulation strategies in ants in comparison to other social insects, with a focus on red wood ants ( Formica rufa group)
Source: F1000Res. 2014 Mar 21;2:280. Originally published 2013 Dec 19. [Version 2] doi: 10.12688/f1000research.2-280.v2 (PMC3962001; doi:10.12688/f1000research.2-280.v2)
Supplement: Peer review history

# Peer review: Referee response for version 2

**DOI:** 10.5256/f1000research.4093.r4237

**Published:** 2014-03-28

**Authors:** Clint Penick

**Affiliations:** 1 Department of Biology, North Carolina State University, Raleigh, NC, USA

The authors have addressed the major points I raised in the previous version.

I have read this submission. I believe that I have an appropriate level of expertise to confirm that it is of an acceptable scientific standard.

# Peer review: Referee response for version 1

**DOI:** 10.5256/f1000research.3163.r3521

**Published:** 2014-03-03

**Authors:** Clint Penick

**Affiliations:** 1 Department of Biology, North Carolina State University, Raleigh, NC, USA

The authors have provided a clear update to the literature on thermoregulation of ant nests and also include interesting anecdotes from the literature, such as Lindauer’s experiment with bees on lava plains. The arrangement of the article focuses on species that regulate temperature by moving brood across a temperature gradient (e.g. *Solenopsis*) vs. species that modify the thermal environment to maintain a core nest temperature (e.g. *Formica polyctena*). This distinction is perhaps the most interesting part of the paper, but in its current form the organization is not clear.

I have the following comments:

1. The authors make their strongest argument for distinguishing between these two strategies in the second to last paragraph. This paragraph should instead be the last paragraph of the Introduction, which would help establish their argument earlier. In addition, several other sections throughout the manuscript could make this distinction more apparent (especially in “ *Behavioural reactions of ant workers*”).
2. It may help to identify which species fit your definitions. *Pogonomyrmex*, *Solenopsis invicta*, *Camponotus*, and *Acromyrmex* fit into brood/fungus movers; *Formica*, *Atta*, *Eciton*, termites, and bees seem to fit more into core nest temperature maintainers.
3. Likewise, the table does not clearly fit within the papers main theme. It may help to add a column that specifies whether each type of nest is associated with brood translocation, maintaining a stable core temperature, or possibly neither.
4. One question that is not addressed in this paper is why these two strategies exist? There is some discussion about wet vs. dry thatch nests in open and forested areas, but I think it would make sense to describe the ecological conditions where each thermoregulation strategy is commonly used in more detail.
5. The discussion about nest ventilation in *Atta *species is not quite accurate. Currently, there is little evidence that nest ventilation strongly impacts temperature; rather, nest ventilation facilitates CO _2_ exchange. This has also been argued with respect to termite nests, where termite nest chimneys do not seem to affect nest temperature.

I have read this submission. I believe that I have an appropriate level of expertise to confirm that it is of an acceptable scientific standard.

# Response

**Published:** 2014-03-19

**Authors:** Štěpánka Kadochová

**Affiliations:** Charles University, Prague, Czech Republic

We thank Clint Penick very much for his review and approval of our review paper. Suggested comments and changes have been included in the latest version of the paper. For cases of authors disagreement with the referee please see comments bellow.

*Point 4  – why those strategies exist?*

Reply: More discussion concerning this topic could be found in part „Use of metabolic heat for nest thermoregulation in ants“

*Point 5 – ventilation in Atta species*

Reply: Authors used information quoted in references, which suppose the ventilation to affect nest temperature.(Kleineidam et. all 2001; Powell and  Stradling 1986, Bollazzi and Roces 2010). However to make situation clear  the sentences about ventilation have been changed to „This ensures exchange of respiratory gasses and optimal thermal conditions for symbiotic fungi which are damaged by temperatures higher than 30°C“ and „Even more efficient ventilation systems, providing both temperature regulation and respiratory gasses exchange, can be found in nests of the termite *Mactorermes bellicose*.“

# Peer review: Referee response for version 1

**DOI:** 10.5256/f1000research.3163.r2859

**Published:** 2014-02-04

**Authors:** Haitao Wu

**Affiliations:** 1 Northeast Institute of Geography and Agroecology, Chinese Academy of Sciences, Changchun, China

This review manuscript represents an interesting study into the various thermoregulation strategies used in ant nests and mounds. A particular strength of the manuscript is the conclusion that there are two main types of thermoregulation strategies used: ‘thermal gradient’ in nests or a ‘stable temperature’ with a heat core. The review concentrates mainly on three aspects to reveal these strategies: nest/mound material and architecture properties, ant behavioral reaction and ant metabolic heat. The manuscript is well written and the discussion seems to be sound. I believe it is a good paper on ant nest ecology, but there are several issues that should be considered.

 

1. Upon reading this paper it is clear that the two different thermoregulatory strategies are its core point, which is explained from three aspects. However these two thermoregulatory strategies are an existing phenomena which are simply being discussed and explored in more detail and should therefore not be the ‘conclusion’ of this article. Instead I think it would be better to state the existence of these two strategies in the Introduction section and then discuses the mechanisms of the phenomena in the rest of the article.
2. Some of the content is rather long and would benefit from being condensed a little to make the overall message clearer. For example: the termites in Table 1 and the details of strategies used by bees, bumblebees and wasps. As this paper focuses on ant thermoregulation characteristics, the content on other insects should deleted as they are not really necessary.
3. The explanations of " *ant metabolic heat*" for ant nest thermoregulation in " *Use of metabolic heat for nest thermoregulation in ants*" are not clear. From the explanations given I still cannot understand how ant metabolic heat impacts on ant mound temperatures or how the ants adopt an ant mound temperature. This content needs to be further explained.
4. The conclusion could do with a rewrite. It should not only reiterate the two opposite thermoregulatory strategies but also condense the messages from other sections, namely: nest/mound material and architecture properties, ant behavioral reaction and ant metabolic heat.

I have read this submission. I believe that I have an appropriate level of expertise to confirm that it is of an acceptable scientific standard.

# Response

**Published:** 2014-03-19

**Authors:** Štěpánka Kadochová

**Affiliations:** Charles University, Prague, Czech Republic

We thank Haitao Wu very much for his review and approval of our review paper. Suggested comments and changes have been included in the latest version of the paper. For cases of authors disagreement with the referee please see comments bellow.

*Point 2 – details of thermoregulatory strategies in other insects*

Reply: The strategies of other social insects species are used as comparism, especialy the bees thermoregulation which is very  well studied. Authrs would like to point out certan paralel between the thermoregulation in Formica polyctena mound to that in bee hive, even though achieved by different mechanisms

*Point 3 – ant metabolic heating*

Reply: The concept of „metabolic heating“ has been described using all known information so far. The metabolic heat production of *Formica rufa* ants has not been studied since the Coenen-Strass study in 1980 so the exact physiological mechanism of metabolic heating has not been clarified yet.

# Peer review: Referee response for version 1

**DOI:** 10.5256/f1000research.3163.r2858

**Published:** 2014-01-22

**Authors:** Jouni Sorvari

**Affiliations:** 1 Department of Environmental Science, University of Eastern Finland, Kuopion, Finland

This is an interesting review on nest thermoregulation strategies in social insects. I think the work is sound and I agree with most parts of the text. However, I think that the authors give too much weight to an assumption that the “dry nest strategy” is a typical strategy for red wood ants. At least in Finland, the large and populous nests are usually located in shaded forests and seem to perform better than the dry nests located in open areas. This is however, overall a good work and deserves to be approved after some fine-tuning.

**Title and Abstract:**

The title expects focus on *Formica rufa*, but in fact the species focused on is *Formica polyctena*, a member of the *Formica rufa* species group. I recommend changing the title so that the focus is on *Formica rufa* group (or “red wood ants”). The Abstract is ok.

**Article content:**

The temperature-humidity regulation of red wood ant nest mounds is a very complex process. Because young small nests of *Formica polyctena* need direct solar radiation to increase the inner temperature of nest, the new nests need to be built in open spots in or close to forests ^1^ ^2^ ^3^. Later, during natural succession those spots will turn into forests, i.e., more shaded habitat but richer in food. The nests will become more shaded but due to the “wet nest strategy” they are no longer dependent on direct sun light. Therefore, both of the two types of thermoregulation strategies are applied in natural populations, but in different stages of nest the development.

The situation would be different if the forest succession did not advance and the nest grows in a spot which stays open for a longer period. This is a typical situation in habitats with anthropogenic influence, where the natural succession and penetration of forest towards agricultural or urban field is prevented. Then the same strategy that is used in shaded forest is not optimal for more permanently open areas. Therefore, the statement that “ *The dry nest thermoregulatory strategy is more common than the wet nest strategy in F. polyctena*” may be true due to anthropogenic pressure, but would possibly not be so in natural habitats. In addition, the statement seems to be based on data studied in two locations in Czech Republic without mention of the naturalness of the habitats. The more fragmented the forests are the higher the proportion of nests that are located in, or close to forest edges and other open areas, possibly causing an impression that the ant prefers open areas.

I recommend using term “moist nest” instead of “wet nest”, because “wet” sounds too wet.

The nest architecture and building method of *Oecophylla* weaver ants is mentioned in the text, but their function in thermoregulation is not mentioned. The authors should either describe how the architecture (or building method) affects thermoregulation of such a nest, or remove the sentence.

Although *Acromyrmex heyeri* builds thatched nests this species does not belong to the ‘thatch ants’ which is in fact an American term reserved for red wood ants of the *Formica* *rufa* group.

**The table:**

Large and most populous nests of the red wood ants, especially the polygynous ones, e.g., *Formica aquilonia* and *F. polyctena*, are often located in shaded forests where direct sun light does not cause high oscillations in outer layers of the nest. It is possible that the description does not fit mature nests in natural forest environment, but young and smaller nests, or nests in anthropogenic fragmented forests.

**References list:**

The reference list should be proofread because of some mistakes e.g., in reference #13 *Formicas. Str*. -> *Formica s. str*.; in some references the species name is not in italics where it probably should be; and the journal abbreviation for Insectes Sociaux should always be Insect Soc.

I have read this submission. I believe that I have an appropriate level of expertise to confirm that it is of an acceptable scientific standard, however I have significant reservations, as outlined above.

## References

[1] PunttilaP: Succession, forest fragmentation, and the distribution of wood ants.*Oikos*.1996;75:291-298Reference source

[2] SorvariJHakkarainenH: Deforestation reduces nest mound size and decreases the production of sexual offspring in the wood ant Formica aquilonia. *Ann Zool Fennici*.2005;48:259-267Reference source

[3] KilpeläinenJPunttilaPFinérLNiemeläPDomischTJurgensenMFNeuvonenSOhashiMRischACSundströmL: Distribution of ant species and mounds (Formica) in different-aged managed spruce stands in eastern Finland. *J Appl Entomol*.2008;132: 280315-32510.1111/j.1439-0418.2007.01244.x

# Response

**Published:** 2014-03-19

**Authors:** Štěpánka Kadochová

**Affiliations:** Charles University, Prague, Czech Republic

We thank Dr. Jouni Sovari very much for his review and valuable comments concerning our review paper. All typos and mistakes have been corrected; suggested comments and changes have been included in the latest version of the paper including the change of title. For comments which need more explanation please see authors replies bellow.

We hope that their explanation and changes made are sufficient enough for the paper version  2  to be accepted.

*JS:* *I recommend using term “moist nest” instead of “wet nest”, because “wet” sounds too wet.*

Reply:  The major reason for using wet nests was to be consistent with previous literature. Term „wet nests“ was adopted because it is how the thermoregulation strategy was described in the previous literature namely Frouz 1996, 2000, Frouz and Finner 2007 . Moreover the moisture of nest material (measured gravimetrically) should reach up to 72%. We believe such level of moisture justify using term wet (15.  Frouz J: The effect of nest moisture on daily temperature regime in the nests of *Formica polyctena*. wood ants. Insec Soc. 2000; 47(3): 229–235.)

*JS: “… Oecophylla weaver ants is mentioned in the text, but their function in thermoregulation is not mentioned …”*

Reply: This is very good note, however the thermoregulatory function of *Oecophylla* nests is not described in any paper so far. We double check that text do not imply any thermoregulation function in these nest. The nest of *Oecophylla* ants were mentioned only to emphasis the great variability of nesting strategies occurring in ants.

*JS – Table 1 „. It is possible that the description does not fit mature nests in natural forest environment, but young and smaller nests, or nests in anthropogenic fragmented forests.“*

Reply: Authors agree with the referee, the thermoreegulatory strategy description is bassed mainly on  limited number of studies performed in Czech republic and in Finland (49. Frouz J, Finer L: Diurnal and seasonal fluctuations in wood ant ( *Formica polyctena*) nest temperature in two geographically distant populations along a south-north gradient. Insect Soc. 2007; 54(3): 251–259). These studies include large variety of nest sizes from nest with volume several litres to volume many thousand of liters. We agree that role of solar radiation is bigger in smaller nests and inner heating is more important in bigger nest and we note this in Table 1

Moreover to make it clear authors mention the possible of different temperation fluctuation in the text as follow: „The seasonal temperature fluctuations ... studied by Frouz & Finer ^49^... both in Finland and the Czech Republic the ant colonies maintained a high nest temperature. Nest temperatures fluctuated more in the Czech Republic than in Finland, possibly because of greater differences between day and night ambient temperatures.“
